# Structural Mechanisms and Drug Discovery Prospects of Rho GTPases

**DOI:** 10.3390/cells5020026

**Published:** 2016-06-13

**Authors:** Cameron C. Smithers, Michael Overduin

**Affiliations:** Department of Biochemistry, University of Alberta, Edmonton, AB, T6G 2H7, Canada; csmither@ualberta.ca

**Keywords:** cancer therapy, signal transduction, GTPase, Rho, RhoA, Rac1, Cdc42, GEF

## Abstract

Rho GTPases regulate cellular morphology and dynamics, and some are key drivers of cancer progression. This superfamily offers attractive potential targets for therapeutic intervention, with RhoA, Rac1 and Cdc42 being prime examples. The challenges in developing agents that act on these signaling enzymes include the lack of obvious druggable pockets and their membrane-bound activities. However, progress in targeting the similar Ras protein is illuminating new strategies for specifically inhibiting oncogenic GTPases. The structures of multiple signaling and regulatory states of Rho proteins have been determined, and the post-translational modifications including acylation and phosphorylation points have been mapped and their functional effects examined. The development of inhibitors to probe the significance of overexpression and mutational hyperactivation of these GTPases underscores their importance in cancer progression. The ability to integrate *in silico*, *in vitro*, and *in vivo* investigations of drug-like molecules indicates the growing tractability of GTPase systems for lead optimization. Although no Rho-targeted drug molecules have yet been clinically approved, this family is clearly showing increasing promise for the development of precision medicine and combination cancer therapies.

## 1. Introduction

### 1.1. Ras Family and Rho Subfamily

The Ras superfamily consists of GTP (guanosine triphosphate)-binding proteins (G proteins) that range in size from 20 to 40 kDa. Most members have an intrinsic guanosine triphosphatase (GTPase) activity that allows them to hydrolyze GTP. They act as binary molecular switches by cycling between an inactive GDP-bound state and an active GTP-bound conformation. When GTP-bound they specifically interact with downstream effectors to stimulate signaling cascades. This enables Ras proteins to regulate cell growth, membrane organization, and nucleocytoplasmic transport [1]. The superfamily is highly conserved in eukaryotes and prokaryotes, with members found in all facets of life [2]. Humans express 167 Ras superfamily members [3], which have been classified into five subfamilies (Ras, Rho, Rab, Sar1/Arf, and Ran) based on structural and functional differences [3]. Here we focus on the Rho subfamily, which offers increasingly attractive targets, particularly for cancer intervention. 

**Figure 1:**
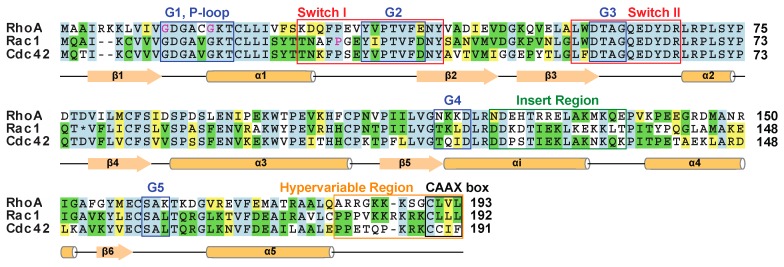
Rho family sequence alignment.

The first Rho family member, Ypt1p, was discovered in yeast in 1985 [4], and identified as a small G-protein [5]. The human genome encodes 22 Rho family members [3] with RhoA, Cdc42, and Rac1 being the best characterized and exemplifying the key functional features (Figure 1). With the exception of eight atypical Rho family members that either bind GTP constitutively (Rnd1, Rnd2, Rnd3, RhoH, RhoU, and RhoV), or are predicted to not bind GTP (RhoBTB1, and RhoBTB) [6], most Rho GTPases are molecular switches which hydrolyze GTP [7]. The various family members are involved in signal transduction pathways that regulate cellular proliferation, apoptosis, polarity, adhesion [1], motility [8], and cytoskeletal organization [9]. Downstream events involving Rho GTPases in response to actomyosin cytoskeleton tensioning include activation of YAP (Yes-associated protein) and TAZ (transcriptional coactivator with PDZ-binding motif) transcriptional regulators [10], as well as nuclear accumulation of MAL (myocardin-related SPF coactivator) and serum response factor (SRF) to activate transcription [11]. The structural mechanisms of RhoA, Rac1 and Cdc42 are particularly well-defined, as are their regulatory partners and post-translational modifications. In light of their emerging roles in cancer progression and the increasing tractability of relatives including Ras for drug discovery, these Rho proteins are discussed here to showcase the development of novel potential GTPase targets for therapeutic intervention.

The amino acid sequences of human RhoA, Rac1, and Cdc42 are aligned with identical, conserved and similar residues highlighted in blue, green and yellow, respectively. The residues that are disease linked are marked with magenta. The asterisks represents a 19-residue insertion found in the Rac1b isoform. Secondary structure elements are underlined and labelled. The sequence alignment was produced using the ClustalW 1.6 similarity matrix [12,13].

### 1.2. The Structure of Rho Family Members

**Figure 2:**
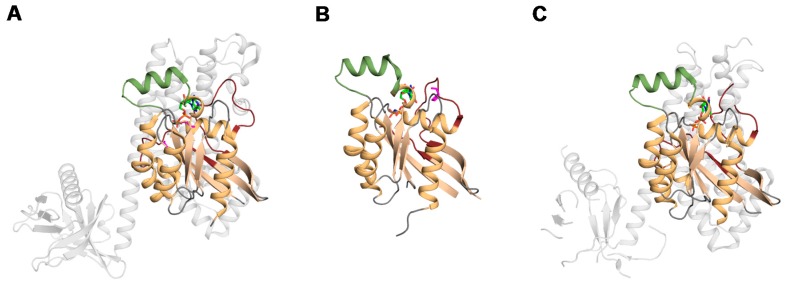
Structures of Rho GTPases. Ribbon structures of A. RhoA (PDB: 4D0N) [33], B. Rac1b (PDB: 1RYH) [29] and C. Cdc42 (PDB: 3QBV) [34] are shown from left to right with the bound regulatory proteins in grey and with mutated sites shown in magenta. The secondary structures and switch elements are labelled and colored as in Figure 1.

The structures of Ras superfamily members reveal a highly conserved core GTPase domain (Figure 2). The GTPase domain consists of several functional features: Five G motifs (named G1 through G5), a core effector domain, and a membrane targeting sequence [14]. The GTPase domains of Rho proteins share 30% identity with other Ras superfamily members, and 40%–95% identity between Rho family members [15]. Numerous structures of Rho GTPases including Rac1, RhoA and Cdc42 have been determined in various functional states. The GTPase domains adopt a Rossmann fold, consisting of a six-stranded mixed β sheet flanked by five α helices [16]. The domain contains two functional elements, switch I and switch II, which undergo subtle conformational changes when GTP binds [17]. The switch I element is core to effector function, with mutations here impairing the interaction with downstream partners [18]. The switch II element has a mechanistic role, coordinating the nucleophilic water that is required for both intrinsic and GAP-mediated GTPase activity [19,20]. Activation of some downstream effectors requires the association with both switch regions. Both switch regions are essential to stabilize the γ-phosphate of GTP within the substrate binding pocket. The G1–G5 loops form the nucleotide-binding site, dictating nucleotide specificity and affinity; ultimately regulating GTP hydrolysis [3]. The G1 motif is located between β1-α1 and is commonly referred to as the phosphate-binding loop (P-loop) due to its pivotal role coordinating the bound nucleotide’s β-phosphate. The P-loop also coordinates a Mg^2+^ ion that is required for nucleotide binding [21]. In addition to nucleotide binding, the P-loop works in conjunction with switch I and switch II to form a binding surface for Rho GAPs [19]. The switch I and switch II regions contribute to the partially overlapping binding sites of many regulators (GEFs, GAPs, and GDIs). The highly conserved mechanism for GDP/GTP exchange among the Ras GTPase superfamily [20] render it difficult to effectively target the active site of disease-associated Rho GTPases for therapeutic intervention, prompting a search for alternative druggable sites.

Rho GTPases have a flexible 20 residue C-terminal extension known as the hypervariable region. This element provides a binding surface for specific downstream effectors and Rho GDIs [21]. Within the hypervariable region is a stretch of adjacent lysine and arginine residues known as the polybasic region. This element forms a unique electropositive patch that forms a binding surface for regulators and effectors [22]. In addition to mediating protein-protein interactions, the polybasic region also mediates association with membranes via interactions with the phospholipid head groups. The membrane targeting sequence includes the C-terminal CAAX box, and occasionally a portion of the hypervariable domain. The CAAX box is conserved in most Rho GTPases, with the exception of RhoBTB and RhoBTB2 [23]. The CAAX box is a tetrapeptide motif (C is a cysteine residue, A an aliphatic residue, and X is any residue) in which the cysteine is post-translationally modified with the addition of a variety covalently linked lipids including farnesyl, or geranyl-geranyl moieties [24]. Within the hypervariable domain and immediately before the CAAX box is a CXXC motif, which is involved in plasma membrane targeting. Some Rho GTPases also undergo covalent addition of a palmitoleic acid group to a cysteine neighboring the C-terminal CAAX box [25]. Once lipid modified, the hypervariable region mediates recruitment of the GTPase to the appropriate membrane [26]. Addition of lipophilic groups to the C-terminus of Rho family members is essential for appropriate subcellular localization and regulation [27].

Comparison of the GTPase structures reveals a unique feature among the Rho family: A helical stretch of approximately 10–15 residues located between α4 and β5 [1,20]. The “Rho insert domain” has no significant impact on the structure in GDP and GTP bound forms [28]. This element modulates Rho’s signaling activity by interacting with GEFs, thus promoting guanine nucleotide exchange. Furthermore, the Rho insert domain assists in perpetuating cell signals by interacting with and activating downstream effectors [21]. Rac1b is a Rac1 splice variant, which contains a distinctive 19 residue insertion immediately after the switch II region. Although the structures of Rac1 and Rac1b are very similar, the insertion and switch I are highly mobile, accelerating GDP/GTP exchange [29]. The atypical RhoBTB and RhoBTB2 relatives contain two long form BTB domains [30]. The first BTB is split by a 115 residue insertion [6], which forms a loop of unknown function. BTB domains mediate protein-protein interactions [31], frequently facilitating the formation of homodimeric [30], heterodimeric [32], and occasionally higher order oligomers [31]. Due to the high degree of overall similarity among both Ras, and Rho family members such subtle structural differences offer opportunities to obtain efficacy and specificity.

## 2. Regulation of Rho Mediated Cell Signaling 

**Figure 3:**
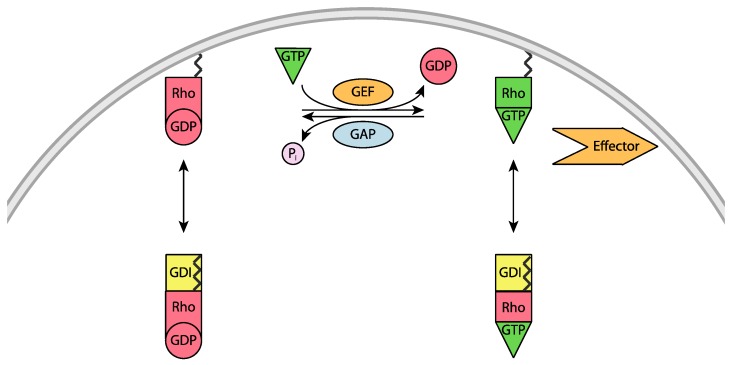
Rho GTPase signaling and the key regulatory proteins. Guanine Exchange Factors (GEFs) activate Rho (green) by mediating the exchange GDP for GTP, while GTPase Activating Proteins (GAPs) promote Rho’s intrinsic GTPase activity, hydrolyzing GTP to GDP and leading to inactivated Rho (red). Guanine Dissociation Inhibitors (GDIs) associate with the covalently bound lipid groups (indicated as jagged black lines) promoting Rho’s dissociation from the membrane. A downstream effectors is coloured orange, and is intended to indicate a range of proteins including ROCKs.

Rho GTPases are key regulators of cytoskeletal structure and dynamics, and influence cell adhesion, morphology and progression through the cell cycle. Hence their cycling between the inactive GDP-bound and active GTP-bound states is tightly regulated, with three types of proteins being centrally involved (Figure 3). Even though cytosolic levels of GTP are greater than GDP, the spontaneous exchange of GDP for GTP is prevented by elevated levels of cytosolic Mg^2+^ [35]. In the presence of Mg^2+^, the binding affinity between small GTPases and guanine nucleotides is in the nanomolar to picomolar range, and dissociation rates are slow, hours in the case of GDP [36]. When external signaling necessitates Rho activation, guanine nucleotide exchange factors (GEFs) promote the exchange of GDP for GTP. The GEFs catalyze nucleotide exchange by reducing the affinity for GDP and transiently stabilizing the nucleotide-free intermediate which can then bind GTP [36]. Rho GTPases self-inactivate via intrinsic hydrolysis of GTP to GDP; however, this occurs at a slow rate. Thus, to inactivate Rho, GTPase-activating proteins (GAPs) accelerate the intrinsic GTPase activity to re-form the GDP bound state. The GAPs reduce the activation barrier for GTP hydrolysis by stabilizing the charged intermediate, and facilitating proper positioning of the hydrolytic water molecule [37]. Finally, the guanine nucleotide dissociation inhibitors (GDIs) are a multifaceted regulators that interact with the inactive GTPase domains and their covalently attached lipid groups. The GDIs prevent the dissociation of guanine nucleotides (typically GDP) from Rho GTPases. When GDIs bind to Rho-GDP, they preserve the GTPase in an inactive state by preventing GEF-mediated nucleotide exchange. When GDIs bind to the GTP bound state, they inhibit GTPase activity and maintaining interactions with downstream effectors; as a result, they maintain a pool of inactive GTP-loaded Rho GTPases. Finally, the interaction between Rho’s lipid moiety and a Rho GDI controls the subcellular distribution of Rho GTPases, cycling between membrane-associated (active) and cytosolic states (inactive) [19]. Cell imaging experiments reveal that over 95% of RhoA is cytosolic [26,38], being sequestered here by Rho GDIs [39]. Together this offers multiple signaling states that could conceivably be targeted.

The Rho family of proteins are both activators and suppressers of ROCK (Rho-associated coiled-coil containing protein kinase) mediated apoptosis [40]. ROCKs are serine/threonine kinases that act downstream of Rho GTPases and mediate the formation of stress fibers and focal adhesions. RhoA, RhoB, and RhoC proteins activate the ROCK pathway by binding to the Rho-binding domain (RBD) of ROCK in their activated, GTP-bound state [41]. In contrast, RhoE promotes cell survival by binding to the N-terminal region of ROCK to prevent the interaction with the RBD and subsequent activation by other Rho family members [42,43]. When active, Rho GTPases modulate nuclear accumulation of factors such as MAL, SRF, TAZ, and YAP to regulate transcription [10,11]. Together this offers multiple signaling states that could conceivably be targeted to alter cellular behaviour.

Numerous post-translational modifications influence Rho activity, with the most-extensively studied being covalent attachment of lipid groups and phosphorylation. Rho family members undergo addition of lipid moieties via prenylation and palmitoylation; adding C-15 farnesyl or C-20 geranyl-geranyl isoprenoid moieties, or a palmitate acyl chain, respectively. Prenylation of the cysteine located within the C-terminal CAAX tetrapeptide motif [27] is necessary for translocation between membranes and the cytosol [35]. The additional hydrophobicity contributed by the prenyl group acts as a membrane anchor [44], but is inserted into a hydrophobic groove found on Rho GDIs when in the cytosolic state [45]. For most Rho family members prenylation is essential for proper function [27], directing their translocation to the appropriate membrane environment [24]; members lacking a prenyl group are misallocated to the cytosol where they are inactive [44]. While not as common, some Rho family members necessitate the covalent addition of a palmitate acyl chain to their C-terminal hypervariable domain found immediately adjacent to the CAAX box [46]. Similar to prenylation, palmitoylation plays a pivotal role in directing these Rho family members to membrane-associated states by increasing the C-terminal hydrophobicity and thus promoting its interaction with the membrane. Prenylation followed by palmitoylation is necessary to direct bCdc42, the brain isoform, to the plasma membrane [47]. Addition of the palmitoyl to bCdc42 is a reversible process, which regulates bCdc42’s association with the membrane [48]; a characteristic found among other Rho and Ras family members [49]. Together, prenylation and palmitoylation play essential roles in directing Rho family members to the appropriate subcellular locale, allowing them to interact with other regulators and downstream effectors.

The second most common post-translational modification to Rho GTPases is phosphorylation. The catalytic domain and regulatory sites that are phosphorylated have been mapped using both site-specific and proteomic-mode mass spectroscopy methods [50], and indicate diverse regulatory mechanisms. Phosphorylation of Rac1 at Ser71 adjacent to switch II by the protein kinase Akt inhibits GTP binding while maintaining its GTPase activity [51]. Phosphorylation of RhoA at Ser188 beside the CAAX box (Figure 1) promotes the association between RhoA-GDP and RhoGDI1 when measured *in vitro*. Formation of the RhoA-GDI complex negatively regulates RhoA activity by enhancing RhoGDI1’s ability to extract RhoA membranes, increasing its abundance in the cytosol while inhibiting RhoA’s ability to hydrolyze GTP. Furthermore, phosphorylation of GTP-bound RhoA alters its ability to interact with downstream; thus directing RhoA activity to certain signaling pathways [52]. Cdc42 is regulated in a similar fashion, where phosphorylation promotes dissociation from the membrane and inhibition by RhoGDI1 [53]. The effectors and regulators of Rho signaling are also phosphorylated, providing a diversity of regulatory influences. The covalent attachment of ubiquitin to lysine residues on RhoA controls its local GTPase activity and mediates its recruitment to the proteasome for degradation, thereby limiting its presence reviewed in [54].

Pathogenic bacteria produce a variety of toxins and virulence factors that covalently modify Rho proteins and alter their activities. These factors alter the ability of Rho proteins to act as molecular switches by mechanisms including adenylylation, ADP ribosylation, deamination, glucosylation, proteolytic cleavage, and transglutamination (reviewed in [54]). For example, the Clostridium difficile toxin B [55] attaches a glucose group to a serine of Cdc42, Rac, or Rho GTPases, thus non-selectively blocking their membrane association and downstream signaling. Another prominent example is the CNF1 toxin from uropathogenic strains of *Escherichia coli* which permanently activates GTPases by deamidating the catalytic glutamine residue in switch-II. Other specific modulators include addition of an AMP molecule or sugar onto a switch-I residue, proteolytic cleave of the C-terminal CAAX box or ADP ribosylation of an asparagine residue in the effector binding region of Rho proteins. While these reactive compounds lack the selectivity needed for development of therapeutic agents, they have proven to be useful for biological studies.

## 3. Role of GTPases in Cancer: RhoA, Rac1, and Cdc42

Ever since the discovery of the ras oncogenes there has been an intimate connection between the Ras superfamily dysregulation and cancer. Given their participation in many cell signaling processes, it comes as no surprise that altered Rho signaling pathways also influence carcinogenesis, and subsequent disease progression. Failure to maintain control of these processes is often the result of either altered levels of gene expression [56], or variations of GDP/GTP exchange. The effects of gene expression levels on tumorigenesis varies among Rho family members and cancer type. Overexpression of Rho GTPases is frequently correlated to cellular transformation, and malignancy. Elevated levels of RhoA expression are observed in numerous forms of cancer, most notably in breast, colon, and lung cancers [57]. Studies of RhoA overexpression in breast cancer cells reveal reduced levels of cell proliferation and invasiveness when treated with RhoA specific siRNAs [58]. Upregulation of Rac1 expression is observed in breast, lung, prostate, and testicular cancer [59,60]. The Rac1b splice variant is overexpressed in colorectal cancer, where in addition to being upregulated, Rac1b is also overactive [29,61,62]. Cdc42 overexpression is observed in breast, colorectal, testicular cancer, and head and neck squamous cell cancer [60,63]. Although overexpression is commonly observed in disease for the well-characterized Rho GTPases, downregulation of some Rho family members has also been linked to cancer [64]. Targeting downregulated proteins is more challenging, and therefore drug discovery efforts have focused on overexpressed and mutant GTPases as the drivers of disease.

In contrast to the Ras GTPase family members, which are mutated in 30% of human tumours [65], cancer-linked mutations are rarely found in Rho GTPase members. Analysis of human tumors has identified a few cancer-linked somatic mutations of Rho GTPases, as listed in the Catalogue of Somatic Mutations in Cancer (COSMIC) database [66]. Cancer-linked point mutations are often found exclusively in a single tissue type and are cancer-specific [67]. Mutation of proline 29 in the switch I region of Rac1 is a hotspot for cancer-linked mutations. Substitution of proline 29 for a serine in Rac1 was observed in 4% to 9% of melanomas [68,69,70]. Functional studies have shown that the P29S substitution reduces Rac1’s GTPase activity by 50%, thereby increasing effector activation [71]. The RhoA G17V mutation to the P-loop is frequently observed in T-cell lymphomas as reported by four groups in 2014 [72,73,74,75]. This abolishes its GTPase activity and as a result RhoA G17V is constitutively active, thereby perpetually activating downstream effectors. Gain-of-function C16R and A161P mutations of RhoA are found in cases of Adult T-cell leukemia/lymphoma, which is caused by the human T-lymphotropic virus type 1 infection [76]. A G12D mutation in P-loop of Cdc42 is observed in melanoma tumors, but its effect on activity is unclear [77]. Together this indicates that Rho mutations found in tumors either constitutively activates Rho, or impairs GTPase activity. 

Cellular transformation is not limited to mutations and upregulation of Rho GTPases; it can be induced by the aberrant control of Rho’s activity by one of its regulators. Elevated expression of Rho GEFs are observed in cancer. Vav1, a Rac1 GEF, is overexpressed in pancreatic adenocarcinoma [78], and metastatic melanoma [79]. A K526Q mutation to the truncated form of AKAP-Lbc, BRX, increases the risk of developing familial breast cancer [80]. Truncation of both the N-terminal and C-terminal regulatory sequences of AKAP-Lbc produces onco-Lbc [81]. Onco-Lbc has constitutively active GEF function [82]. The onco-Lbc protein induces cancer in a Rho dependent manner [83]. The LARG (leukemia-associated Rho GEF) protein mediates activation of RhoA and signals for bleb-associated cancer evasion [84]. Reducing Rho activation by targeting the RhoGEF complex formation has garnered interest among various groups interested LARG/RhoA [85] and AKAP-Lbc/RhoA [86] (P.C) Designing therapeutics which target the protein-protein interfaces between Rho GTPases and their regulators are likely to provide specificity, thus representing attractive potential targets.

Deregulated Rho GTPases can promote the transformation, survival, and migration of cancer cells [87]. The Rho family members influence tumorigenesis through transcription factor activity and regulating gene expression that ultimately alters cell cycle progression, increasing cell proliferation and reducing apoptosis. The Rho GTPases are known modulators of the transition from G_1_ phase to S phase. The activity of a few Rho-proteins influence the expression levels of cyclin D1, a major integrator of G_1_ signaling, with elevated levels initiating DNA synthesis [88]. RhoA, Rac1, and Rnd3 are all known stimulators of cyclin D1 expression [89]. Integrin specific activation of Rac1 is able to simulate cell cycle progression of endothelial cells through G_1_ [90]. Rac1 is also essential in Ras mediated transformation of NIH-3T3 cells [91]. Alterations in RhoD expression are known to cause centrosome duplication, and mediate the transition from G_1_ to S via mDia1 (mammalian homolog of the *Drosophila* diaphanous protein) [92]. The mDia1 protein acts as an effector of a small Rho GTPase to regulate actin cytoskeleton remodeling via interactions with the acting polymerizing protein profiling 1. RhoE prevents the dissociation of translation initiation factor eIF4E from 4E-BP1, thereby inhibiting translation of cyclin D1 and Myc, arresting the cell cycle in G_1_ [93]. In addition to instigating uncontrolled proliferation, Rho GTPases promote cancer cell survival influencing both pro- and anti- apoptotic signaling pathways. As key regulators of cell cycle progression and apoptosis, changes to the activity of Rho-proteins have a profound effect on protein expression and activity which leads to uncontrolled proliferation.

In addition to promoting tumorigenesis, altered Rho GTPases also contribute to disease progression through cell signaling pathways which regulate cell adhesion, cell motility, metastasis, and angiogenesis. The common theme among these processes is the necessity for the dynamic reorganization of the actin cytoskeleton. For epithelial cells to maintain their polarity, cell-to-cell contact must be maintained. Various animal models have shown the activity of RhoA, Rac1 and Cdc42 is indispensable. The activities of RhoA and Rac1 are essential for cadherin-mediated cell-cell adhesion in keratinocytes. Cells treated with C3 transferase, a RhoA inhibitor, or overexpressing dominant negative Rac1 (N17), are unable to form cell-cell contacts [94]. E-cadherin induced polarity of rat kidney epithelial cells (NRK-52E) cells requires Cdc42 activity [95]. Cdc42 deficient mice have cell polarity defects [96]. Loss of cell polarity is a hallmark of cancer [97], with the loss of epithelial cell polarity being common among carcinoma calls [87]. Invasive epithelia cancer cells have reduced cell-cell adhesion and display increased motility [98]. Once cellular adhesion is lost, the probability of cancerous cells invading the neighboring tissues or metastasizing increases [99]. Individual cancers cells are able to invade neighboring tissue by either mesenchymal or amoeboid migration [100]. During mesenchymal migration the activities of Rac1 and Cdc42 drive the leading edge forwards, whereas RhoA signals the contraction of the trailing edge [101]. Amoeboid motion occurs by cycles of expansion and contraction of the cell body by altering the distribution of actin and myosin [102]. Amoeboid motion is regulated by the RhoA/ROCK pathway, which controls the actomyosin contractility [103]. Both A375m2 melanoma and LS174T colon carcinoma cells display a Rho- and ROCK-dependent amoeboid-like motion [104]. For tumor cells to metastasize to distant tissues they must access either the vascular or lymphatic system [105]. In order to metastasize, cancerous cells use the Rho/ROCK pathway to cross the endothelial lay and enter the bloodstream or access the lymphatic system [15]. The activity of Cdc42 is important for metastasis of PC-3 cells and thus represents a potential therapeutic target [56]. SiRNA silencing of RhoA in PC-3 and MDA-MB 231 cells yielded a reduced migration speed and a net reduction of movement [106]. MDA-MB 231 derived tumors treated with RhoA and RhoC specific siRNA marked a significant reduction in tumor volume [58]. Inhibiting of certain Rho GTPases may reduce the ability of cancerous cells to invade new tissues, and prevent secondary tumor formation by inhibiting the mechanism which allow for tumor metastasis. Once a tumor has formed, neovascularization of the cancerous tissue is essential for cancer progression. The process of angiogenesis requires alterations in cell permeability, extracellular matrix, cell migration, proliferation, and survival. *In vivo* and *in vitro* studies have identified Rho proteins as essential in signaling for vascular endothelial growth factor (VEGF)-dependent capillary formation [107]. The process of angiogenesis assists cancer progression by improving cancer cell survival and metastasis. Increased tumor vasculature supports tumor growth by increasing access to oxygen and nutrients, while removing waste products [108]. Tumors with increased vascularity correlate with poor patient prognosis [109]. Conversely, insufficient vascularization of a tumor can result in the tumor becoming necrotic [110] or apoptotic [111]. Once the primary tumor is vascularized, the malignant cells now have access to the circulatory system, allowing them to metastasize to distant organs. Targeting Rho GTPase activity to prevent the neovascularization of cancerous tissue would reduce and hereby limit tumor size.

## 4. Targeting Rho GTPase Signaling for Therapeutic Intervention

The discovery that Rho family GTPases are overexpressed, mutated and deregulated in several forms of cancers has motivated the search for inhibitors. Recent successes with the discovery of novel pockets and inhibitors of Ras [112] have further emboldened the field. Nonetheless, targeting the GTP binding site directly remains inherently challenging due to its picomolar nucleotide affinity and the high concentrations of GDP and GTP in cells. The potential has been demonstrated by a variety of virulence factors that have been isolated from pathogenic bacteria and nonspecifically target the Rho family of proteins [54], spurring the design of selective noncovalent agents. Recent efforts have focused on exploiting novel pockets and the heterogenous interfaces involved in selective GEF interactions. For example, ITX3 has been identified as a selective small molecule inhibitor of the N-terminal GEF domain of Trio and blocks activation of Rac1 and RhoG [113].

Small molecules that specifically inhibit Rac and Cdc42 have been designed to compete with GEF and effector interactions. Rac family members are inhibited by EHT1864, which causes nucleotide release and prevents effector binding, thus disrupting lamellipodia formation [114]. Virtual screening for ligands of the Trp58-containing GEF interface yielded a selective Rac1 inhibitor [115]. This compound, NSC23766 (secramine), shows biological activity in prostate cancer cell lines, and a structure of its Rac1 complex guided further optimization [116] and design of a inhibitor with cellular activity at 1 μM [117]. Structures of the complex A selective Cdc42 inhibitor, CASIN, was identified by cell based assays and binds the GTPase surface in such a way as to specifically block the interaction of its GEF intersectin [118]. Nucleotide analogs have been designed for Cdc42, and act as noncompetitive allosteric inhibitors that induce ligand dissociation and reduce filopodia formation and cell migration [119]. A more selective Cdc42 inhibitor, ML141, was identified by high-throughput screening using fluorescent GTP and appears to bind to an allosteric site of gunanine-nucleotide bound GTPase, inducing ligand dissociation and inhibiting neurite outgrowth [119]. Virtual ligand screening yielded ZCL278 as a selective Cdc41 inhibitor, which appears to suppress actin-based cellular functions such as Golgi organization by disrupting binding of GTP and its GEF intersectin [120].

Inhibition of the RhoA-family GTPases is also of increasing interest, with structures of the relevant GEF complexes emerging [33,82]. Virtual ligand screening of 4 million compounds yielded a 0.4 μM affinity Rho inhibitor named Rhosin. It binds to the surface of RhoA by Trp58, suppresses interaction of the LARG GEF-stimulated and blocks RhoA-mediated cytoskeletal activity and invasiveness of breast cancer cells [121]. A fluorescent ligand-based screen of ten thousand compounds yielded five selective inhibitors of LARG-stimulated RhoA-GTP binding [122]. An inhibitor that binds between the DH and PH domains of the LARG GEF was identified by virtual screening, and selectively blocks RhoA signaling and tumorigenic behavior of breast cancer cells [123]. Surface plasmon resonance analysis of hits from virtual screening yielded validated ligands that inhibit Rho, thus inducing vasorelaxation in thoracic aorta artery rings [124]. NMR-based screening of a thousand drug fragments identified a ligand that binds near the RhoA switch II region and inhibited the interaction with the LARG GEF, thus constituting a novel starting point for the design of selective inhibitors [85]. A quinoline series has been explored, resulting in an inhibitor that binds specifically to RhoA with 1 μM affinity and shows efficacy for cardiovascular disease in cell models [125]. Further complexed structures and structure activity relationships are emerging for Rho family members, and strategies to target related GTPases are being adopted. Together this will provide valuable tool compounds to better understand the critical target states and pockets, and may be used to optimize lead candidates for clinical trials. Together this demonstrates the growing potential of targeting Rho GTPases and their regulatory complexes for therapeutic intervention.

## 5. Conclusions

The design of therapeutic agents that target Rho family members is increasingly feasible based on their increasingly well-defined roles in cancer. Future exploitation of this family will benefit from the wealth of crystal structures of target states and off-target GTPases, and the advent of fragment-based drug discovery that allows identification and exploitation novel druggable pockets. Modern NMR capabilities including ultra-high field magnets, cryogenic probes and robotic autosamplers provide enhanced resolution, sensitivity, and throughput for rapid analysis of ligand binding, allosteric and regulatory mechanisms of Rho proteins and their partners. This approach uniquely provides atomic-level information of the structure, dynamics and interactions of proteins including GTPases on membrane mimicking bicelles and lipid discs that are important for activation *in vivo*. In combination with complementary techniques including surface plasmon resonance, this allows the rapid development of hits into specific lead molecules with efficacy in cancer cells. The ability to identify transient pockets, stabilize transitory target states and exploit protein-protein interfaces is converting previously undruggable proteins into increasingly tractable targets, as underscored by progress with Ras superfamily members. Here we have highlighted three established Rho family targets, which have already yielded new pockets and leads for optimization into drug molecules. This is opening the door for discovery of chemical probes and inhibitors of GTPases and their partners, setting the stage for clinical testing of lead candidates based on understanding of detailed mechanisms of action.

## Abbreviations

The following abbreviations are used in this manuscript:

GAP	GTPase Activating Protein
GEF	Guanine nucleotide Exchange Factor
GDI	GDO Dissociation Inhibitor
LARG	Leukaemia-Associated Rho GEF
AKAP	A-Kinase Associated Protein
mDia1	Diadphanous-related formin-1
eIF4E	Eukaryotic translation Initiation Factor 4E
4E-BP1	Eukaryotic translation initiation factor 4E Binding Proteim-1
ROCK	Rho-associated protein Kinase
VEGF	Vascular Endothelial Growth Factor
